# Plant optics: underlying mechanisms in remotely sensed signals for phenotyping applications

**DOI:** 10.1093/aobpla/plad039

**Published:** 2023-07-06

**Authors:** Christopher Y S Wong

**Affiliations:** Department of Plant Sciences, University of California, Davis, Davis, CA, USA

**Keywords:** Ecophysiology, machine learning, phenology, phenotyping, pigments, remote sensing, solar-induced fluorescence, stress, vegetation indices

## Abstract

Optical-based remote sensing offers great potential for phenotyping vegetation traits and functions for a range of applications including vegetation monitoring and assessment. A key strength of optical-based approaches is the underlying mechanistic link to vegetation physiology, biochemistry, and structure that influences a spectral signal. By exploiting spectral variation driven by plant physiological response to environment, remotely sensed products can be used to estimate vegetation traits and functions. However, oftentimes these products are proxies based on covariance, which can lead to misinterpretation and decoupling under certain scenarios. This viewpoint will discuss (i) the optical properties of vegetation, (ii) applications of vegetation indices, solar-induced fluorescence, and machine-learning approaches, and (iii) how covariance can lead to good empirical proximation of plant traits and functions. Understanding and acknowledging the underlying mechanistic basis of plant optics must be considered as remotely sensed data availability and applications continue to grow. Doing so will enable appropriate application and consideration of limitations for the use of optical-based remote sensing for phenotyping applications.

## Teaser

Interested in using optical-based remote sensing for plant phenotyping? This viewpoint discusses the mechanistic link between leaf physiology and spectral signatures to enable applications of vegetation indices and machine-learning models for monitoring and assessing plant status.

## Introduction

**Figure 1.**
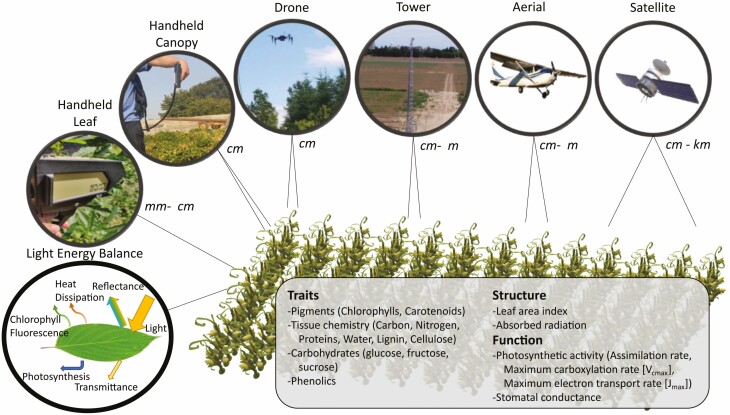
Optical-based remote sensing captures reflectance providing insight based on the optical properties of vegetation. This enables potential for quantifying vegetation traits, functions, and structure to infer vegetation health and status (examples listed in the box). Sensors can be deployed on various platforms with respective spectral, temporal, and spatial resolutions.

Plant phenotyping represents tools that can be used to quantify vegetation traits, structure, and function and respective interactions with the environment (Fiorani and Schurr 2013; Watt *et al.* 2020). The ability to phenotype vegetation is important for many applications including the evaluation of physiological and biochemical traits, stress responses, growth and yield, and parameterization of terrestrial ecosystem models (Furbank and Tester 2011; Janni and Pieruschka 2022). However, many plant phenotyping methods are labour intensive and time-consuming making large-scale and continuous monitoring impractical. Remote sensing offers a powerful tool that can complement ground-based methods in meeting plant phenotyping needs (Chawade *et al.* 2019; Machwitz *et al.* 2021). An advantage of remote sensing is that it can be deployed across a suite of platforms that covers a range of spatial and temporal scales potentially enabling high-throughput phenotyping capabilities (Fig. 1). At the finest spatial scale, handheld devices can be used to measure individual leaves with a leaf clip or to measure individual plants and canopies from short distances (e.g. 1 m). For larger spatial coverage, sensors can be mounted on ground-based platforms including vehicles, towers, and gantry systems (Gamon *et al.* 2006; Virlet *et al.* 2016; Xu and Li 2022; Wong *et al.* 2023*b*). There are also aerial platforms including aircraft, balloons, and unpiloted aerial vehicle (UAV) systems (Chen and Vierling 2006; Zarco-Tejada *et al.* 2012; Chapman *et al.* 2014; Guo *et al.* 2021; Wang *et al.* 2022*a*). Finally, at the largest spatial scale, there are satellite platforms (Zhang *et al.* 2020*a*). This paper will focus on optical-based remote sensing, defined as reflected or re-emitted energy between 400 and 2500 nm. Note that there are other types of remote sensing systems for plant sciences as well, which include thermal (Pineda *et al.* 2021; Farella *et al.* 2022), LiDAR (Lin 2015; Jin *et al.* 2021), and radar (Steele-Dunne *et al.* 2017; Orynbaikyzy *et al.* 2019).

Optical-based remote sensing captures the amount of re-emitted or reflected radiation from a surface relative to the total incoming irradiance from a light source (e.g. sun or lamp). Depending on surface optical properties influencing how light is absorbed, transmitted, and reflected across the visible and near-infrared (NIR) spectrum, distinct spectral patterns can be attributed to land surface characteristics (e.g. vegetation, soil, snow, water, etc.). For vegetation monitoring, reflectance spectra are sensitive to leaf biochemistry (i.e. pigments and macronutrients), water content, and structure (Ustin *et al.* 2004). This enables a mechanistic link between spectral reflectance and vegetation properties. Thus, remote sensing products can be used to quantify a suite of plant traits and functions (Fu *et al.* 2020). Consideration of spectral data in relation to the underlying mechanistic optical properties, and respective response to environment is needed during phenotyping applications. This paper will contribute to existing in-depth reviews on the relationship between spectral reflectance and leaf biochemistry (Ustin and Gamon 2010; Cavender-Bares *et al.* 2017; Jacquemoud and Ustin 2019; Kothari and Schweiger 2022), but focus on phenotyping applications in plant sciences. Specifically, this paper will discuss (i) vegetation optical properties, (ii) applications of vegetation indices, solar-induced fluorescence (SIF), and machine-learning approaches, and (iii) considerations of covariance and limitations for phenotyping. For both current or prospective users of remote sensing technologies in plant sciences, this viewpoint aims to describe the mechanistic basis of plant optics to help users determine their needs and provide direction into future remote sensing research and applications.

## Optical Properties of Vegetation

**Figure 2.**
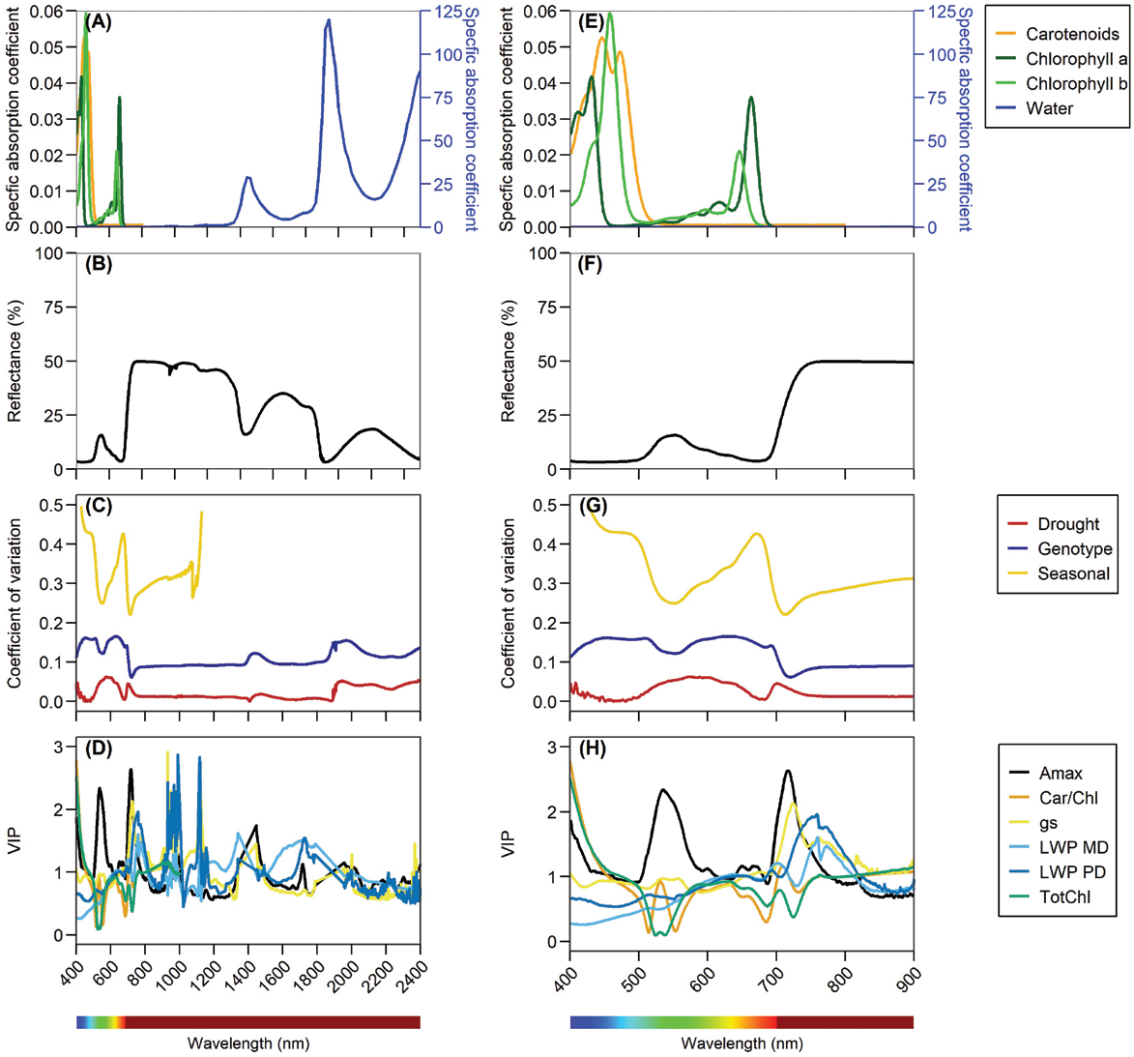
Examples of leaf pigment and water specific absorption coefficients (A, E), leaf spectral reflectance (B, F), spectra coefficient of variation across different scenarios (C, G; drought response between irrigated and terminal drought common beans, genotype differences across a population of 300 common beans, seasonal response of lodgepole pine over two years), and variable importance in projection (VIP) from partial least squares regression models for plant physiology variables (D, H; Amax: maximum assimilation rate, Car/Chl: carotenoid/chlorophyll pigment pool ratios, gs: stomatal conductance, LWP MD: midday leaf water potential, LWP PD: predawn leaf water potential, TotChl: total chlorophyll a and b pools). Left panels show full range spectra from 400 to 2400 nm, and right panels show visible and near-infrared regions from 400 to 900 nm. Data sources: pigments (Clementson and Wojtasiewicz, 2019), water (Hale and Querry, 1973) and spectra (Wong and Gamon, 2015; Wong *et al.* 2019, 2023a).

The physiological and structural properties of vegetation may affect vegetation optical properties across different regions of the spectrum (Gates *et al.* 1965; Knipling 1970). By understanding light absorption and reflectance, spectra can be used to infer physiological, structural, and biochemical features of vegetation. In addition, depending on the sampling context of vegetation such as across genotypes, species, and time (diurnal, weekly, seasonal, interannual), the degree of spectral variation will vary, which can be represented by the coefficient of variation of reflectance values (standard deviation relative to the mean) at each wavelength. The visible region of the spectrum (400–700 nm) is highly sensitive to light absorption by pigments, especially the chlorophylls, carotenoids, and anthocyanins (Blackburn 2007). Chlorophylls (a and b) absorb strongly in the blue and red spectral regions, while carotenoids absorb strongly in the blue region (Fig. 2E), and anthocyanins absorb strongly in the green region (Gitelson *et al.* 2001). Carotenoids are a widely distributed group consisting of pigments such as xanthophylls (violaxanthin, antheraxanthin, and zeaxanthin), lutein, and alpha- and beta-carotene (Maoka 2020). Chlorophylls and carotenoids are generally present in leaves across their developmental stages, whereas anthocyanins may not be present in all plants, and generally only in very young developing leaves or senescing leaves (Hoch *et al.* 2001; Steyn *et al.* 2002). Therefore, with chlorophylls and carotenoids being the most dominant pigments strongly absorbing light, typical healthy green leaves have distinct spectral features in the visible region with low reflectance in the blue and red regions due to high light absorbance (Fig. 2E and F). There is also a distinct reflectance peak in the green region due to lower light absorption relative to the blue and red region by chlorophyll a and b, and a sharp increase in reflectance beyond 700 nm, often called the red edge, where chlorophylls and carotenoids do not absorb light. Since the visible spectral region is strongly influenced by pigments, this region often shows high variation driven by pigment composition and pool size, relative to the rest of the spectra (Fig. 2G). During short-term stress events (e.g. drought, temperature, and light), variation is observed in the blue-green region where the highly dynamic xanthophyll pigments are (violaxanthin, antheraxanthin, and zeaxanthin). Here, carotenoid interconversion via the xanthophyll cycle occurs on the timescale of seconds to minutes, which is often linked to non-photochemical quenching and light-use efficiency (Bilger and Björkman 1990; Demmig-Adams 1990). Across genotypes (and species), spectral variance is high in the blue and red regions driven by variations in chlorophyll and carotenoid pigment composition and pools (Wright *et al.* 2004). There is also high spectral variance across seasons, which encompasses leaf growth, maturity, and senescence—or for evergreen conifers, spring recovery and winter downregulation—with the highest variance in the blue, red, and red edge driven by major changes in chlorophyll and carotenoid pools (Ottander *et al.* 1995; Wong and Gamon 2015). Because pigments represent variation across a range of stress responses and phenological changes, the visible region offers powerful potential in assessing vegetation physiology and function tied to the roles of chlorophylls and carotenoids in absorbing and dissipating light energy (i.e. the light energy balance) (Hüner *et al.* 1998; Sims and Gamon 2002). This is highlighted by relatively high spectral variation, indicated by the coefficient of variation, in the visible spectrum across genotypes and environmental conditions (Fig. 2C and G).

Beyond the visible spectral region, is the NIR region (700–1100 nm), where pigments do not absorb light. Here, because there are no strong absorption features, most light is reflected or transmitted. Light transmission through a leaf is impacted by characteristics like leaf thickness, intracellular space compactness, and membrane thickness (Asner 1998). For example, with thicker leaves and/or more compact leaves, transmittance will be lower—leading to higher reflectance (Knapp and Carter 1998). Since leaf structure represents a physical process, the NIR region generally highlights higher spectral variance across genotypes and phenology (Fig. 2C). In contrast, there is minimal structural variation within leaves during short events like stress indicated by lower spectral variation since leaves rely more on immediate pigment dynamics than rearranging internal leaf structure. However, there may be physical leaf changes from wilting or leaf movement impacting canopy NIR reflectance, detectable at the canopy scale (Gamon and Pearcy 1989; Pastenes *et al.* 2005; Sapes *et al.* 2022).

The shortwave infrared (SWIR) spectral region (1100–2500 nm) is also sensitive to leaf structure in addition to other absorption features. The most pronounced features are water absorption within a leaf near 1450, 1950, and 2500 nm (Allen and Richardson 1968; Gao and Goetz 1994) (Fig. 2A and C). Thus, when leaf water content decreases, reflectance at these features will increase. However, the use of these water absorption features is confounded at remote distances due to atmospheric water absorption in the same spectral regions. Due to this, canopy-based measurements from tower, airborne, and satellite systems avoid or filter out data in these spectral regions. Physiologically, the SWIR region is also sensitive to biochemical (e.g. nitrogen, protein, lignin, and cellulose) (Curran 1989) and phenolic compounds (Kokaly and Skidmore 2015).

Variation of leaf spectra enables the inference of vegetation traits. These traits, in turn, vary between species and genotypes, in response to environment over the short- (sub-daily to weeks) and long-term (weeks to seasons), and local conditions such as management or resource availability. Therefore, by evaluating variation across full spectral data or in specific spectral regions, information about leaves and canopies can be utilized to infer vegetation dynamics spatially and temporally.

## Applications of Plant Optics

Remotely sensed quantification of plant traits often uses approaches that exploit wavelengths associated with physiological and structural properties, thereby providing a mechanistic link between spectra and the estimated trait. Depending on instrument specifications, there will be constraints on spectral, spatial, and temporal range and resolution. Spectral range represents the portion of the spectrum that is observable. Spectral resolution represents the number of bands available and respective bandwidths (i.e. full-width half maximum [FWHM]). Therefore, both spectral range and resolutions for a given sensor can impact which plant optical properties can be observed. The most widely adopted sensors are often multispectral in spectral resolution, covering only a select few bands typically at coarser FWHM bandwidths. The wavebands can sometimes be selected for specific applications to suit user needs. The other type of sensor is hyperspectral in spectral resolution, which is often more expensive compared to its multispectral counterpart, covering full range spectra from the visible and NIR (~400 to 1100 nm) and sometimes including the SWIR (~1100 to 2500 nm) at relatively high spectral resolution (<3 nm; i.e. finer FWHM and high number of bands). Thus, hyperspectral sensors offer the most flexible applications enabling the visualization and use of the full spectral dataset to evaluate a suite of vegetation traits (as shown in Fig. 2). Ultimately, by accounting for user needs and sensor specifications, several approaches have been developed to quantify plant traits by exploiting variation in spectral reflectance.

Vegetation indices provide a simple approach by utilizing a few select wavebands to exploit a mechanistic optical signal. Many vegetation indices have been developed to quantify chlorophyll content (Peñuelas *et al.* 1995*a*; Gitelson *et al.* 1996, 2003; Gitelson and Merzlyak 1997; Datt 1999). Chlorophyll is relatively easy to detect because of the strong absorption signal (Fig. 2A), thereby enabling vegetation indices to exploit reflectance in the red or red edge where chlorophylls solely and strongly absorb light. Quantifying carotenoids is relatively more difficult because of the overlapping absorption feature with chlorophyll in the blue region. As a result, vegetation indices for carotenoid content attempt to use a narrow waveband near 500 nm yielding limited performance (Blackburn 1998). Instead, the carotenoid:chlorophyll ratio is generally more easily assessed by normalizing carotenoid absorption with chlorophyll absorption bands (Gamon *et al.* 2016; Gitelson 2020). For quantifying macronutrients and phenolic contents, approaches exploit spectral reflectance in the SWIR, which is directly sensitive and avoids overlapping absorption features with pigments. However, in the SWIR region leaf and atmospheric water absorption must be carefully considered, which may limit the wavebands available for use. Leaf nitrogen concentration demonstrates high correlation with 2054 and 2172 nm linked to absorption characteristics of N-containing amide bonds (Kokaly 2001). For phenolics, reflectance demonstrates high correlation near 1660 nm linked to C–H bonds (Kokaly and Skidmore 2015). In both these studies, quantification using these wavebands generally performs better with dry leaf material compared to fresh leaf material due to the overlapping water absorption features. Alternatively, vegetation indices have also been developed to empirically estimate biochemical compounds like nitrogen content and Rubisco using visible and red-edge wavebands by leveraging a tight link with chlorophyll content (Tarpley *et al.* 2000; Cho and Skidmore 2006; Feng *et al.* 2014; Magney *et al.* 2017).

Beyond estimating pigments, macronutrients and phenolic content, vegetation indices have also been applied as proxies of vegetation physiology and ecosystem functions. One of the most common vegetation indices is the normalized difference vegetation index (NDVI), based on red and NIR reflectance (Tucker 1979). NDVI has been used as a proxy of light absorption (i.e. fraction of absorbed photosynthetically active radiation [fAPAR]) and leaf area index (LAI) (Myneni and Williams 1994; Gamon *et al.* 1995; Carlson and Ripley 1997; Myneni *et al.* 2002; Fensholt *et al.* 2004). Here, NDVI has shown to perform well in applications as a vegetation stress indicator and for monitoring phenology (Pettorelli *et al.* 2005; Huang *et al.* 2021). Since NDVI has served as a basis for vegetation monitoring, efforts have also sought to expand on NDVI by minimizing the influence of background signals (e.g. soil, understory) and LAI saturation via the enhanced vegetation index (Liu and Huete 1995; Huete *et al.* 2002; Jiang *et al.* 2008), soil-adjusted vegetation index (Huete 1988), and NIR reflectance of vegetation index (NIRv) (Badgley *et al.* 2017). This has led to applications of vegetation indices being proxies of photosynthesis (i.e. gross primary productivity) (Sims *et al.* 2006, 2008; Badgley *et al.* 2019; Baldocchi *et al.* 2020). For photosynthetic activity, carotenoid sensitive vegetation indices have also shown promise, especially in evergreen conifer systems where NDVI has limited seasonal variability due to chlorophyll retention (Sims *et al.* 2006; Walther *et al.* 2016; Magney *et al.* 2019; Pierrat *et al.* 2022*b*). These vegetation indices take advantage of carotenoid pigments and their association with photoprotection (Demmig-Adams 1990), thereby providing a more robust indicator of photosynthetic activity beyond chlorophyll content (Gamon *et al.* 1997; Garrity *et al.* 2011). For example, the photochemical reflectance index (PRI) exploits spectral variation near 531 nm, driven by carotenoid pigment interconversion via the xanthophyll cycle (Gamon *et al.* 1992; Peñuelas *et al.* 1995*b*). Because of the functional role of carotenoid pigments in photoprotection dynamics, PRI has been used as a proxy of light-use efficiency, non-photochemical quenching, and photosynthetic activity (Garbulsky *et al.* 2011; Zhang *et al.* 2016). The chlorophyll/carotenoid index, an analogue of PRI, exploits phenological variation of chlorophyll:carotenoid pigment ratios to perform as a proxy of phenology and photosynthetic activity (Gamon *et al.* 2016; Wong *et al.* 2019). For estimating plant water concentrations, vegetation indices exploit water absorption features in the NIR and SWIR (Hunt and Rock 1989; Penuelas *et al.* 1997; Ceccato *et al.* 2001). Overall, there exists a vast number of vegetation indices for various applications in monitoring vegetation; here only a few major vegetation indices were highlighted. Overall, vegetation indices may serve as simple and powerful proxies of vegetation traits and functions with a mechanistic link for respective applications (Zeng *et al.* 2022).

Recently, remote sensing retrievals of SIF have opened a pathway for a more direct measure of chlorophyll fluorescence emissions to serve as an indicator of photosynthetic activity (Frankenberg *et al.* 2011; Joiner *et al.* 2011; Porcar-Castell *et al.* 2014, 2021). To retrieve a SIF signal, specialized instruments with very high spectral resolutions (~0.3 nm FWHM) in the red or far-red regions are needed to employ the Fraunhofer line depth principle to differentiate SIF from reflected radiation (Plascyk 1975; Meroni *et al.* 2009; Mohammed *et al.* 2019). A key strength of SIF is that it is based on a re-emitted signal from chlorophylls. Because of this, SIF represents a more physiologically direct signal linked to the light reactions of photosynthetic activity, resulting in a strong relationship to gross primary productivity across many environmental conditions (Sun *et al.* 2023*a*, *b*). In contrast, vegetation indices are based on light reflectance driven by variation in pigment pools to serve as proxies of physiology and function, which can be prone to decoupling depending on environmental conditions (Bannari *et al.* 1995). In phenotyping applications, SIF has demonstrated great potential for assessing photosynthetic performance of vegetation across different environmental conditions (Camino *et al.* 2019; Fu *et al.* 2021; Krämer *et al.* 2021; Wang *et al.* 2022*a*; Wong *et al.* 2023*b*).

Beyond the use of specific wavebands for vegetation indices, full hyperspectral data may be utilized to exploit subtle variations across the visible, NIR, and SWIR to predict plant traits and functions. This often requires complex analysis such as machine-learning models with physiological validation data for model calibration. Different machine-learning approaches have been utilized including principal components analysis, spectral decomposition analysis, support vector machine, random forest, convolutional neural networks, and partial least squares regression (PLSR), all of which have performed well (Cheng *et al.* 2020; Hennessy *et al.* 2020; Burnett *et al.* 2021; Zhang *et al.* 2021; Pierrat *et al.* 2022*a*). These machine-learning approaches utilize different algorithms with respective assumptions, data transformations, and model fittings to weigh spectral importance in a predictive model. For plant trait and function predictions, PLSR is often used due to its simpler approach and outputs of the model spectral weights via variable importance in projection (VIP), which is useful for interpretability of spectral features. As machine learning uses full hyperspectral data, it considers information from all spectral regions and respective leaf optical properties to enable model prediction for specifically calibrated plant traits and functions. For example, like vegetation indices, hyperspectral data has been used to predict foliar pigment and macronutrient content such as chlorophyll, carotenoid, nitrogen, protein, and carbon content (Feng *et al.* 2008; Zhang *et al.* 2008; Serbin *et al.* 2014; Singh *et al.* 2015; Zhao *et al.* 2016; Ely *et al.* 2019; Sonobe *et al.* 2020; Shi *et al.* 2022). Models, such as PLSR, have also been calibrated to predict plant functions such as maximum carboxylation rate (*V*_cmax_), maximum electron transport rate (*J*_max_), stomatal conductance, and water potential (Silva-Perez *et al.* 2018; Wu *et al.* 2019; Meacham-Hensold *et al.* 2020; Wong *et al.* 2023*a*). These models have demonstrated generally good performance with respect to calibration for detecting variation across several environmental conditions including drought, nitrogen deficiency, and phenology and between species and genotypes—highlighting promise in remotely sensed phenotyping applications.

The ability of spectra via vegetation indices and full hyperspectral analysis to phenotype vegetation traits and functions enables potential applications in areas such as research, management, and plant breeding (Sishodia *et al.* 2020; Guo *et al.* 2021; Ustin and Middleton 2021). In addition to phenotyping traits, spectral information can also be used for discriminant classification. For example, species identification may exploit species-specific spectral differences based on pigment pools and structure for producing species identity maps (Adam *et al.* 2010; Li *et al.* 2021). Within species, applications may include disease detection based on physiological and structural response of vegetation to disease, ultimately reflected by spectra (Zhao *et al.* 2016; Zarco-Tejada *et al.* 2018; Gold *et al.* 2020; Zhang *et al.* 2020b; Calamita *et al.* 2021; Hornero *et al.* 2021). Genotypic responses to environment may also be captured to help identify stress-tolerant genotypes (Sinha *et al.* 2020; Chai *et al.* 2021; Crusiol *et al.* 2021; Wong *et al.* 2023*a*). Beyond phenotyping applications, spectra may also provide insight into biodiversity and trait evolution (Cavender-Bares *et al.* 2016, 2017; Meireles *et al.* 2020).

## Covariance and Considerations

Optical-based remote sensing offers great potential for phenotyping applications. However, much work remains to advance these techniques for reproducibility, interpretation, and reporting for robust applications. Ultimately, these challenges emphasize the importance of understanding the underlying properties contributing to optical signal as well as respective limitations. A major aspect of optical remote sensing is covariance between observable vegetation traits and less observable plant traits and functions, because of the lack of a direct observable spectral feature (Wright *et al.* 2004; Kokaly *et al.* 2009; Ollinger 2011). As discussed in Section “Optical Properties of Vegetation”, the main spectral features are pigments (chlorophyll and carotenoids) in the visible, leaf and canopy structure in the NIR, and water absorption in the SWIR regions. Thus, using certain combinations of spectral features, vegetation indices offer a way to predict plant traits and functions directly and indirectly (proximally). Direct predictions are typically limited to pigments because of their role in light absorptance (Gitelson *et al.* 2001, 2003; Gitelson 2020). However, remote quantification of pigments still relies on empirical relationships, which could limit wider use spatially and temporally across different vegetation types. Even widely used vegetation indices are prone to limitations due to decreased sensitivity and saturation. For example, NDVI is often used as a proxy of LAI or absorbed radiation because of covariation with chlorophyll content (Myneni and Williams 1994; Gamon *et al.* 1995; Carlson and Ripley 1997). Yet despite this, NDVI may saturate in dense ecosystems (i.e. high LAI) because strong light absorption by chlorophylls leads to minimal variation in red reflectance and thus decreases NDVI’s sensitivity (Sellers 1985; Gitelson *et al.* 2003). Vegetation indices linked to plant function like PRI performed as a proxy of light-use efficiency because of covariation with the xanthophyll cycle (Gamon *et al.* 1992; Peñuelas *et al.* 1995*b*). However, across seasons, the underlying mechanisms driving the PRI signal shifts to reflect the more dominant changes in canopy structure and chlorophyll:carotenoid pigment pool ratios (Sims and Gamon 2002; Wong and Gamon 2015). This may limit general use because of varying empirical relationships dependent on spatial, temporal and vegetation dynamics. SIF bypasses the limitation of relative reflectance signals since SIF captures chlorophyll fluorescence emissions in radiance units, which will not saturate even in high canopy chlorophyll content systems (Porcar-Castell *et al.* 2021; Sun *et al.* 2023*a*, *b*). However, since chlorophyll fluorescence (and SIF) only represents one of three pathways for quenching absorbed light energy (Maxwell and Johnson 2000; Baker 2008), there may be decoupling between fluorescence and photosynthetic activity during periods of high stress (e.g. drought and heatwave) when photochemical and non-photochemical quenching pathways become limited or saturated (Porcar-Castell *et al.* 2014; Magney *et al.* 2020; Maguire *et al.* 2020; Marrs *et al.* 2020; Martini *et al.* 2022; Pierrat *et al.* 2022*b*).

Machine-learning approaches, while taking advantage of the full spectrum, offer a more ‘direct’ prediction of plant traits and functions via model calibration, but these approaches remain tied to covariance. A useful output is the variable importance in projection (VIP) from PLSR, which can identify the spectral weighing of a model (Fig. 2D and H). Often, the visible spectral region is an important factor, because of the roles and high variance of chlorophyll and carotenoid pigments, leading to strong covariation with many vegetation functions (Fig. 2D and H). The NIR region generally shows relatively lower VIPs because it represents leaf and canopy structure, which is generally less dynamic than pigments when looking at a single species as is the case in Fig. 2. In some instances, structure may have a role in capturing phenology and developmental stages (Baldocchi *et al.* 2020; Noda *et al.* 2021) or during stress events that lead to wilting (Sapes *et al.* 2022). The inclusion of the SWIR region in machine-learning models is less explored because of instrument limitations in spectral range. Recent studies have shown that the addition of SWIR leads to negligible improvement in predicting photosynthetic parameters and biochemical content (Ely *et al.* 2019; Meacham-Hensold *et al.* 2020). For traits linked to plant water status like water potential and stomatal conductance, SWIR may improve model performance (Junttila *et al.* 2022; Wong *et al.* 2023*a*). Machine-learning approaches enable use of the full spectrum, which can maximize variation to predict plant traits and functions. This often leads to high covariation of predicted plant traits and functions with pigments because of how dynamic pigments are in response to environment. Beyond environmental response, pigment composition and pool size may vary across genotypes, species, developmental stages, and leaf age. Because of a machine-learning model’s sensitivity to pigment covariation for predicting traits and functions, much work is needed to explore general applications and performance of models across species, local site conditions, and time.

In addition to underlying mechanisms influencing vegetation optical properties, consideration of external influences of spectra must also be considered. This is especially important since a key component of remote sensing is to measure vegetation from a distance to enable more practical applications. At remote distances, atmospheric water vapour absorption overlaps with leaf water absorption features, requiring careful consideration of the SWIR region (Gao 1996; Sims and Gamon 2003). Canopy structure may also influence spectral properties due to light transmission and reabsorption within a canopy. This may lead to background signals from understory and soil contributing to a spectral signal (Colwell 1974). Canopy structure is an important consideration for physiological-driven indicators such as PRI (Hilker *et al.* 2008; Yang 2022) and SIF (Biriukova *et al.* 2020; Porcar-Castell *et al.* 2021). There may also be physical attributes from sun-sensor geometry influencing a spectral signal, but this influence may be minimized by keeping observations near solar noon and at nadir sensor positions or applying bidirectional reflectance distribution function corrections (Schaaf *et al.* 2002; Jacquemoud *et al.* 2009; Hao *et al.* 2022). Despite these challenges, their impacts on spectral signal may be minimized with standardized data collection protocol. Machine-learning approaches and some vegetation indices may inherently account for some of the canopy structure influences due to the inclusion of NIR wavebands for normalization (Zarco-Tejada *et al.* 2013; Wang *et al.* 2017). SIF has also seen improvements upon consideration of canopy structure (Braghiere *et al.* 2021). Ultimately, many of these considerations and their effects on optically phenotyping traits and functions must be further explored to gain insight into the robustness of remote sensing-based phenotyping for general applications.

Despite the challenges for the general use of remote sensing in plant phenotyping, there lies great potential to advance our understanding of remotely sensed signals to improve phenotyping applications. As vegetation indices rely on empirical relationships and machine-learning approaches require calibration, their robustness and broad applicability remain a question. This includes how these models perform across (i) years; (ii) environmental conditions (e.g. water and nutrient availability); and (iii) other vegetation types. Experiments are often performed in controlled conditions inducing single stress. In these conditions, plant optics has demonstrated its ability to phenotype stresses including drought (Sun *et al.* 2015; Wong *et al.* 2023*a*), disease (Gold *et al.* 2020; Calamita *et al.* 2021), and nutrient deficiencies (Feng *et al.* 2008; Ely *et al.* 2019). However, while stress detection is possible, identifying the stress source in uncontrolled conditions proves a major challenge (Lassalle 2021). Environmental stress regardless of the source (e.g. drought, nutrient, disease) will induce plant responses in general, thus complicating stress identification. Perhaps with hyperspectral data calibrated to predict a suite of plant traits and functions, this could advance stress detection via the exploitation of how specific plant traits and functions respond to certain stresses (Ghosal *et al.* 2018; Singh *et al.* 2018). Well-controlled and/or large-scale studies will be needed to address these questions. Furthermore, predicting plant traits and functions will only provide information on the status of the plant, and not necessarily whether the plant response is ultimately beneficial. This will require further information such as growth, biomass, yield, or fitness to help assess the full extent of the plant response, especially for plant breeding and agricultural needs. It is also important to tease apart differences in plant response due to management strategy versus changes in plant growth stage. This is especially true for vegetation indices which provide a relative assessment of vegetation status. Thus, including control plants (e.g. irrigated, nutrient-rich, disease-free) will assist in quantifying the response of vegetation under varying conditions (Christie *et al.* 2020; Rogers *et al.* 2021).

An advantage of optical-based remote sensing is that it can be used to assess plant traits and functions. This leads to complementary applications with other remote sensing products including thermal and LiDAR and for extrapolating ground-based measurements to larger spatial and temporal scales. For example, thermal data provides information about canopy temperature and is often used to assess evapotranspiration (Chen and Liu 2020). Combining evapotranspiration data with indicators of photosynthetic activity (e.g. SIF) could lead to insight on water use efficiency (Cai *et al.* 2021). LiDAR enables the 3D measurement of canopy structure to assess structural information such as biomass, plant size, and leaf angles. This information could provide complementary details on plant structure with trait/functional information from plant optics (Lin 2015; Jin *et al.* 2021). In addition, leaf angles could help correct and improve spectral quality by accounting for variation driven by leaf geometry (Behmann *et al.* 2016). For complementing ground-based measurements, which are labour intensive (i.e. time and personnel), remote sensing offers spatial and temporal extrapolation. Ground-based measurements could be performed less frequently or intensively (e.g. measuring a subset). With a subset sampling scheme (e.g. measure 10% of plots), ground data could be used to calibrate remotely sensed vegetation indices or machine-learning models and the remaining plots outside of the subset could be remotely assessed, assuming good and representative model calibration. Overall, while optical-based remote sensing techniques enable applications in plant phenotyping, it also provides complementary aspects with other remote sensing techniques and more traditional plant science methodologies.

## Conclusions and Outlooks

The applications of optical-based remote sensing continue to grow for monitoring and assessing vegetation in ecology, plant sciences, and precision agriculture. A vast amount of spectral data continue to be collected, improved (spatial and temporal resolutions), and made more accessible. Recent and future satellite missions including Carbon Mapper, Copernicus Hyperspectral Imaging Mission For The Environment (CHIME) (Nieke and Rast 2018), Earth Surface Mineral Dust Source Investigation (EMIT) (Green *et al.* 2020), and Surface Biology and Geology (Cawse-Nicholson *et al.* 2021) will provide spectral and thermal imagery at frequent temporal (weekly to bi-weekly) and high spatial resolutions (<30 m). This will provide ample opportunities for vegetation monitoring and revolutionize the potential for large-scale and continuous phenotyping applications. Yet with all the streams of data, there remains a need to validate and understand the mechanisms driving spectral signals. Ultimately, optical-based remote sensing models will often be based on covariance between pigments and structure to estimate plant traits and functions (Wright *et al.* 2004; Kokaly *et al.* 2009; Ollinger 2011; Kothari and Schweiger 2022; Wang *et al.* 2022*b*). This has implications for how robust predictions can be across years, species, locations, and environmental conditions. Thus, understanding how vegetation pigments and structure vary spatially, temporally, and environmentally will provide insight into prediction robustness. By predicting several plant traits and functions, optical-based remote sensing products may complement each other by providing information about many processes to gain a better overall understanding of vegetation status. Combined with other tools, such as thermal, LiDAR, and ground measurements, there is a great potential to complement, benefit, and advance applications in ecology, plant sciences, management, and plant breeding.

## Data Availability

Spectral data from Wong and Gamon (2015) and Wong *et al.* (2019, 2023a) and available upon request.

## Notes

**Plants, Ecosystems and Climate. Chief Editor:** Mary Heskel
